# The Biomechanical Mechanism of Upper Airway Collapse in OSAHS Patients Using Clinical Monitoring Data during Natural Sleep

**DOI:** 10.3390/s21227457

**Published:** 2021-11-10

**Authors:** Liujie Chen, Tan Xiao, Ching Tai Ng

**Affiliations:** 1 School of Civil Engineering, Guangzhou University, Guangzhou 510006, China; cechenliujie@gzhu.edu.cn; 2 Center for Mechanical Teaching and Testing, Guangdong University of Petrochemical Technology, Maoming 525000, China; 3 School of Civil, Environmental & Mining Engineering, The University of Adelaide, Adelaide, SA 5005, Australia; alex.ng@adelaide.edu.au

**Keywords:** obstructive sleep apnea hypopnea syndrome, natural sleep, monitoring device, one-way valve effect, biomechanical simulation, individualized treatment

## Abstract

Obstructive sleep apnea hypopnea syndrome (OSAHS) is a common sleep disorder characterized by repeated pharyngeal collapse with partial or complete obstruction of the upper airway. This study investigates the biomechanics of upper airway collapse of OSASH patients during natural sleep. Computerized tomography (CT) scans and data obtained from a device installed on OSASH patients, which is comprised of micro pressure sensors and temperature sensors, are used to develop a pseudo three-dimensional (3D) finite element (FE) model of the upper airway. With consideration of the gravity effect on the soft palate while patients are in a supine position, a fluid–solid coupling analysis is performed using the FE model for the two respiratory modes, eupnea and apnea. The results of this study show that the FE simulations can provide a satisfactory representation of a patient’s actual respiratory physiological processes during natural sleep. The one-way valve effect of the soft palate is one of the important mechanical factors causing upper airway collapse. The monitoring data and FE simulation results obtained in this study provide a comprehensive understanding of the occurrence of OSAHS and a theoretical basis for the individualized treatment of patients. The study demonstrates that biomechanical simulation is a powerful supplementation to clinical monitoring and evaluation.

## 1. Introduction

In 2020, the number of OSAHS cases was about one billion. The direct and indirect diagnosis and treatment costs caused by OSAHS are challenges for the global healthcare system [1,2]. Obstructive sleep apnea hypopnea syndrome (OSAHS) is associated with coronary heart disease, pulmonary heart disease, hypertension, diabetes, hypercapnia, and cognitive impairment, and can cause stroke, acute myocardial infarction, or sudden death [3,4]. OSAHS is potentially linked to the emerging pandemic as it may increase the risk of death from COVID-19 [5]. The pathogenesis of OSAHS is presently unclear. It is commonly recognized that OSHAS is caused by structural abnormalities in the upper airway and its surrounding tissues, flaccidity of the pharyngeal muscle during sleep, and collapse of airway soft tissue [6,7]. Polysomnography (PSG) is considered the gold standard for diagnosing OSAHS [8,9]. A variety of monitoring technologies, such as portable home sleep breathing monitors, upper airway and esophageal pressure sensors, three-dimensional upper airway computerized tomography (CT) scans, and magnetic resonance imaging (MRI), are also used to screen and assist in the diagnosis of OSAHS. In the literature, there has been no validated analytical model for accurately explaining and predicting the mechanism of collapse of the soft tissues, such as the soft palate, corpus linguae, uvula, and epiglottis. In general, the mechanisms of upper airway blockage have been studied using mechanical models.

Compared with traditional experimental methods, biomechanical simulation has the advantages of being noninvasive, highly manipulative, and reiterative. It allows an emphasis on individuality and reflection of individual patient conditions [10,11]. This research area has attracted significant interest. Research work has focused on formulating relevant mechanical models of the upper airway and its surrounding tissues, and studying the respiratory process and movement with the assistance of high-performance computers and commercialized numerical analysis software [12,13,14]. Currently, there are three challenges related to numerical simulation of OSAHS:

- Lack of clinical data on OSAHS patients in natural sleep for model construction

In most of the current case studies, CT data collected from awake patients or healthy people [15,16] were used. The data of sleeping subjects are typically obtained during sleep induced by anesthesia [17,18]; however, it has been clinically found that a majority of OSAHS patients are able to breathe normally in an awake state, even patients with severe OSAHS, who only suffer from the collapse of the upper airway in the sleep state [19,20].

Abnormalities in the anatomic structure of the upper airway can be modelled by changing the geometric boundaries of the finite element (FE) model. It can be observed that certain morphological changes occur in the upper airway due to diminished neural control of the dilatation muscles of the upper airway during sleep. Under this condition, the area and degree of stenosis of the upper airway differ from those observed in the awake state, and some differences can also be observed from initial geometric boundaries.

To improve the accuracy of prediction, it is essential to model the natural sleep state using FE simulations. In this paper, CT data obtained from a 33-year-old male patient with severe OSAHS in the natural sleep state is used for biomechanical modeling.

- Lack of clinical monitoring data for modelling boundary conditions

The boundary conditions of the inlet and outlet of the airway have a significant impact on numerical results. Conventionally, researchers used to choose a flow value at the inlet, and assign a velocity or pressure boundaries at the outlet in OSAHS simulations [15,21]. The disadvantage of assigning such boundary conditions is that the entire airflow volume of the respiratory simulation process becomes a fixed value.

Airflow is caused by a pressure difference between the inlet and outlet of the airway, regardless of whether the OSAHS patient is sleeping in a eupnea or apnea state. If the boundary conditions of the model are defined based on certain subjective assumptions, errors or even distortions may occur in the simulation results.

In this study, the boundary conditions derived from the curve of the pressure change, which was measured clinically in the laryngeal cavity, are applied in the FE simulations of both eupnea and apnea. In the simulations, the volume of the entire airflow changes according to the conditions of the airflow. This provides a better model for a patient’s physiological process of respiration.

- Obscurity caused by airway collapse and the phase

Most previous studies reported that airway collapse occurs in OSAHS patients during the inhalation phase of the respiratory process. Remmers et al. [22] asserted that airway collapse takes place in the inhalation phase and that it can be explained by elevated negative pressure in the upper airway. Gold and Schwartz [23] found that it is difficult for the pharyngeal cavity of OSAHS patients to resist the relatively high pressure from surrounding tissues; thus, the ability to sustain an open airway is insufficient.

In contrast, Yucel [24] reported that the retropalatal and retroglossal regions of both healthy people and OSAHS patients have smaller cross-sectional areas in the exhalation phase than in the inhalation phase. Other studies [15,25,26,27] reported that collapse of the airway occurs at the end of the exhalation phase; however, the mechanism causing the collapse is still unclear. Morrell et al. [25] suggested that the lung volume of OSAHS patients under sleeping conditions decreases at the end of expiration, making their upper airways more prone to collapse. Zhu et al. [15] simulated the movement of the soft palate of an individual under normal breathing conditions using a fluid–solid coupling technique. They found that the movement of the soft palate during expiration is much greater than that during inspiration. The continuous decrease in the volume of the retropharyngeal cavity due to the movement of the soft palate towards the retropharyngeal wall could trigger a blockage of the airway at the end of expiration.

We conducted a number of observations using clinical magnetic resonance (MR) of OSAHS patients. It was found that airway collapse and blockage are more likely to occur in patients whose soft palates are hypertrophic, hyperplastic, or flaccid. The anatomic structure of the soft palate resembles a one-way valve. Although this allows smooth intake of air by the patient during inspiration during sleep, it confines the outbound airflow by closure of the valve section when the interior pressure of the cavity reaches a threshold level during expiration.

In consideration of the three aforementioned challenges, there are four focuses in this study. (1) Carrying out clinical experiments, such as dynamic CT scans, on the OSAHS patient in a natural sleep state, and monitoring the interior cavity pressure of the airway during eupnea and apnea. (2) Development of a quasi-three-dimensional (3D) FE model of the OSAHS patient’s upper airway and its surrounding tissues based on the CT data. The FE model focuses on the sagittal plane with a 10-mm thickness preserved on the coronary plane. (3) Determination of the outlet boundary conditions using the pressure values of the pharyngeal cavity obtained from the clinical monitoring sensors while the patient is sleeping under eupnea or apnea. The effects of gravity on the soft palate are taken into account for the patient while in a supine position. (4) Carrying out FE simulations with fluid–solid coupling to simulate the airway collapse during a complete cycle of respiration when the OSAHS patient is in a natural sleep state. The interaction between the internal fluid field of the patient’s airway and its surrounding tissues under the two different breathing modes (eupnea and apnea) was investigated. This verifies the clinically observed one-way valve effect of the soft palate and makes it possible to investigate the pathogenesis of OSAHS from a mechanical perspective. The insights gained from this study could advance clinical diagnoses and the personalized treatment of OSAHS.

## 2. Data and Methods

### 2.1. Data

#### 2.1.1. Clinical CT Data and Establishment of Finite Element Model

The medical imagery used in this research was retrieved from a 33-year-old Asian patient with OSAHS. The patient had a snoring history of two decades, and was degree III on the Friedman scale. Thus, a diagnosis of severe OSAHS with moderate hypoxemia was confirmed.

**Figure 1:**
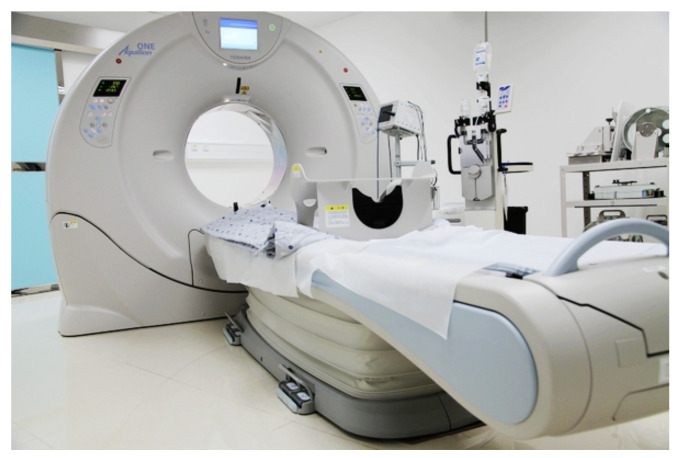
Toshiba Aquilion ONE 640-slice dynamic organ volume CT (taken in the Radiology Department, the Sixth Affiliated Hospital of Sun Yat-sen University).

**Figure 2:**
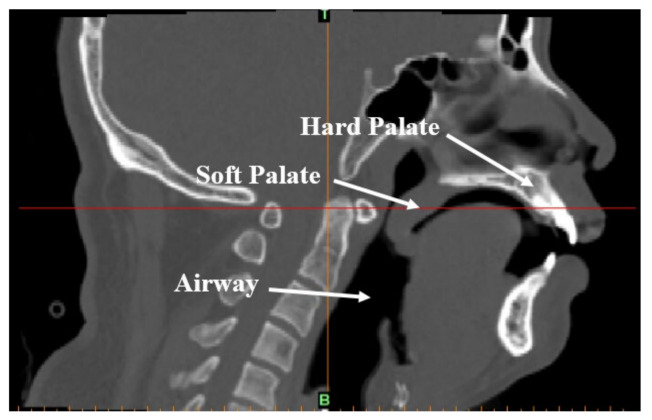
CT-scanned image of the upper airway of an OSAHS patient in a supine position and sleeping naturally.

For minimizing the time in putting the patient to natural sleep for the scan, and eliminate the effect of tranquilizing drugs, the patient was required to stay awake for the entire night prior to the experiment and avoid consuming beverages or taking medication that was naturally exciting or tranquilizing before the examination. The patient was expected to sleep naturally in a supine position before the CT scan could commence. When the patient began to snore, the medical staff called the patient’s name gently and the patient did not wake up, confirming the patient was in a state of deep sleep. A Dy-volume scan from the basis cranii to the cricoid cartilage was performed on the patient using a Toshiba Aquilion ONE 640-slice dynamic organ volume CT scanner (Toshiba, Tokyo, Japan) to obtain the CT data of the patient’s upper airway during natural sleep, as shown in Figure 1. The scan time was set to 10 s and the scan thickness was 0.5 mm. When the scan was completed, DICOM format images were obtained and are shown in Figure 2.

**Figure 3:**
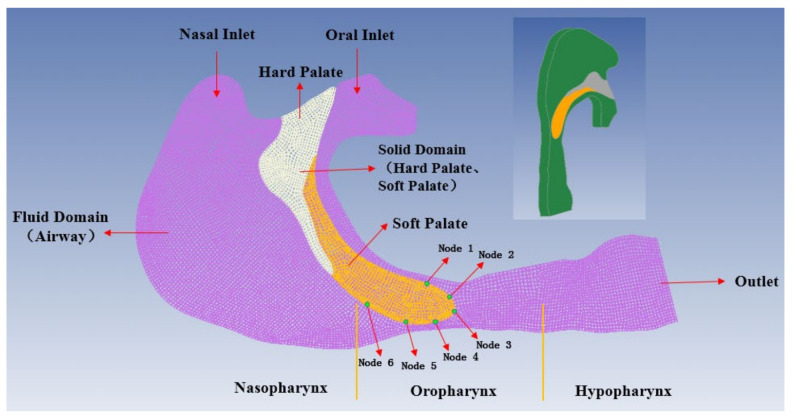
The FE model of the fluid field of the upper airway, along with the solid fields of the soft and hard palates.

After simplification of the data obtained from the nasal tissue, a quasi-3D FE model of the upper airway and its surrounding tissues was constructed and is shown in Figure 3. The thickness of the FE model was 10 mm. There were 88,693 nodes and 77,390 elements for the upper airway fluid field, 9240 nodes and 10,130 elements for the soft palate, and 8349 nodes and 6840 elements for the hard palate. Hexahedral elements were adopted in the solid and fluid domains of the upper airways, addressing with the incompressibility problem with a full integration formulation. In the model, the solid domain of the hard palate was set as the fixed boundary. The airway was modelled as fluid, and the junction between the airway and the soft palate was modelled by coupling the surfaces.

#### 2.1.2. Clinical Monitoring of Cavity Pressure

**Figure 4:**
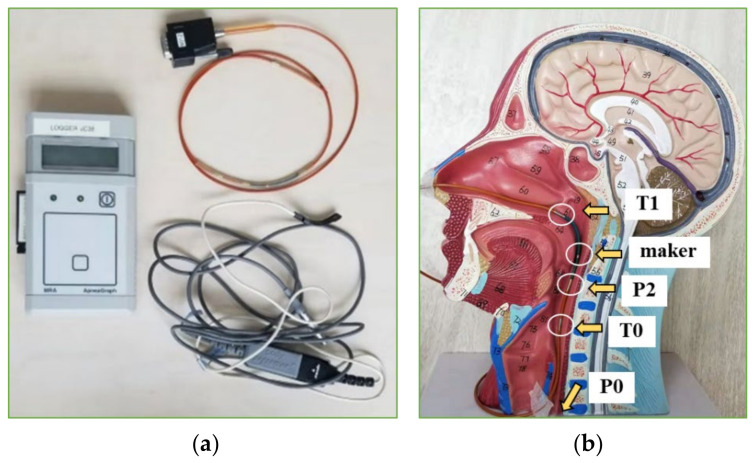
Airway pressure fluctuation monitoring (taken in the Sleep-Disordered Breathing Center, the Sixth Affiliated Hospital of Sun Yat-sen University). (**a**) ApneaGraph AG 200; (**b**) schematic diagram of upper airway pressure monitoring.

The patient was monitored using ApneaGraph AG 200 (MRA-Medical Ltd., Gloucestershire, UK), as shown in Figure 4, for 6–8 h at night, and comprised micro-pressure and temperature transducer catheters. The procedures were to first sterilize the piezometer tube with glutaraldehyde, then select one nostril of the patient, which was comparatively unobstructed, and administer aerosols of ephedrine followed by tetracaine to the selected nostril and oropharynx. With the anesthesia administered and after a 10 min wait, tube insertion operation began by inserting the piezometer tube via the anaesthetized nostril. When the metal marker was at the highest level of the free end of soft palate, the pressure sensors, P0 and P2, were positioned at the mid-section of the esophagus and at the oropharynx beneath the free edge of soft palate; thermal sensors T1 and T0 were located in the nasopharynx and hypopharynx, respectively.

**Figure 5:**
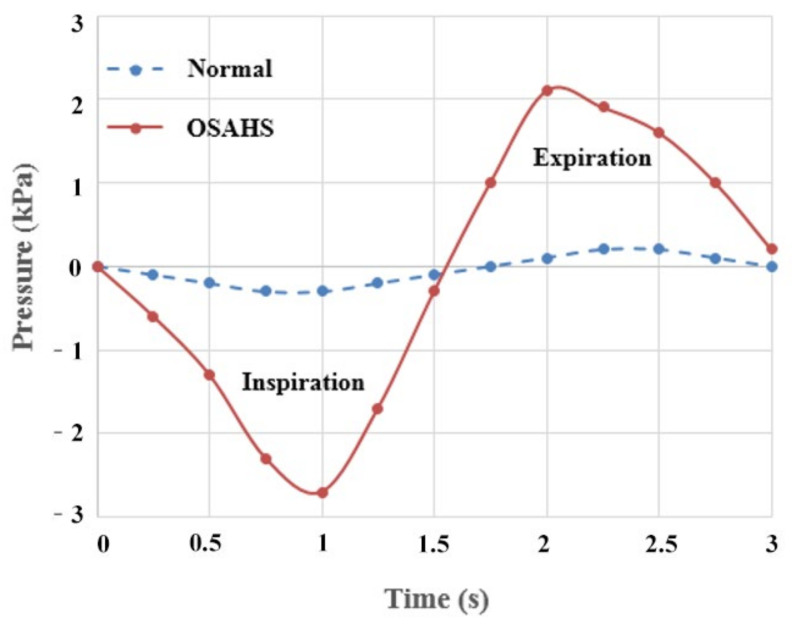
The time-history curves of cavity pressure of the OSAHS patient during sleep.

Pressure data obtained during the typical 3-s respiration cycle in cases of eupnea and apnea are shown in Figure 5. The red solid line denotes the pressure in apnea while the blue dashed line denotes the pressure in eupnea. The markers denote the data obtained from the clinical pressure measurement from the sensor. The clinically measured pharyngeal pressure from the ApneaGraph AG 200 was used to assigned the boundary condition at the outlet of the upper airway in the FE model. At the airflow inlet (the nasal cavity of the model), a pressure of 1 atm was exerted. All respirations mentioned in the processes above refer to nasal respirations.

In the FE models, all the tissues were modelled using linear constitutive relation. The tissues in the soft palate contain a considerable amount of water, which is an incompressible material.

The mechanical properties of the estimated soft palate are very sensitive to the adopted methodology and individual variations [28]. For instance, the Young’s modulus of a soft palate determined was found to vary from 0.5 kPa to 1000 kPa [29,30,31]. In a previous study, movements of the soft palate under different Young’s modulus values were simulated, which indicated that the simulation result of obstruction and one-way valve effect were close to those observed under clinical MR when the Young’s modulus of the soft palate was 1 MPa. Predictably, if the Young’s modulus of the soft palate is smaller, obstruction will be more pronounced.

In this paper, the Young’s modulus of the soft palate was set as 1 MPa and a Poisson’s ratio of 0.49 was used; the density was 1000 kg/m^3^. The Young’s modulus of the hard palate was set as 13.7 GPa and a Poisson’s ratio of 0.3 was used; the density was 1850 kg/m^3^. The medium in the fluid field was air and the density and dynamic viscous coefficient were 1.7894 × 10^−5^ kg/ms and 1.225 kg/m^3^, respectively. In addition, the upper airway was treated as an instantaneous rigid body and the fluid was considered incompressible. The change in the temperature field during the simulation was neglected, and the effect of gravity on the soft palate in the supine position was considered. A complete respiration cycle was simulated.

### 2.2. Methods

#### 2.2.1. One-Way Valve Structure of the Soft Palate

A one-way valve is a structure that maintains a single direction of fluid flow at a given location in a fluid passage. This device comprises one or multiple relatively stationary pivots or bases, in which motion change is a function of the direction of the fluid flow. The pivot of the one-way valve in the soft palate is located at its intersection with the hard palate.

**Figure 6:**
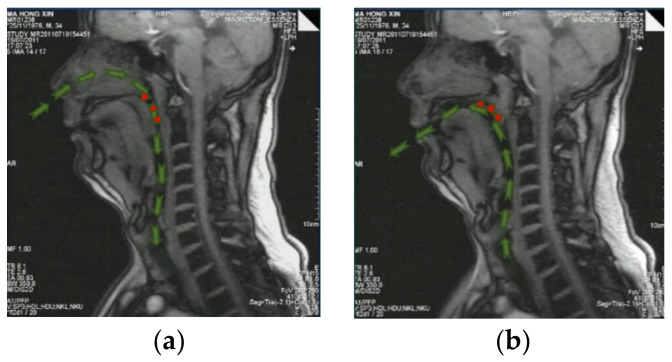
One-way valve effect of an OSAHS patient. (**a**) Act of inhaling; (**b**) exhaling. Green arrows indicate the airflow path.

When a patient with OSAHS inhales through the nose, air flows through the valve area and causes snoring. During the expiration phase, the valve area in the soft palate would be closed, and hence, there is no nasal passage for the exiting airflow and the air is forced to exit from the mouth. In our experiment, the one-way valve effect of an OSAHS patient was clinically observed by means of high-speed dynamic MR, as shown in Figure 6.

#### 2.2.2. Fluid-Solid Coupling and Arbitrary Lagrangian Eulerian Algorithm

In an OSAHS event, collapse of the airway is the result of an interaction between the fluid field (the airflow in the airway) and the solid field (the surrounding airway tissues). Among the various methods used for OSAHS in the literature, FE with fluid–solid coupling [32] is the most commonly used approach [33,34].

The Arbitrary Lagrangian Eulerian (ALE) algorithm was implemented using the commercial software ANSYS/LS-DYNA for fluid–structure interaction analyses of the upper airway. The fluid region was described using the ALE method, the Lagrange method was used to describe the solid region, and the fluid–structure interaction was defined by the penalty coupling algorithm. The deformation exhibited by the collapse of the soft tissue typically exceeds the range defined as a small deformation. If the magnitude of the structural deformation is too large, it could lead to a severe distortion in the FE mesh that can stall or even abort numerical simulations. The ALE algorithm can overcome the computation difficulties caused by the distorted elements and facilitate the dynamic analysis of the fluid–solid coupled system. The ALE includes two overlapping meshes, a spatial mesh that is allowed to move randomly in space, and a mesh that is attached to the material of interest and moves with the material within the spatial mesh. Given the incompressibility and viscosity of the time-dependent airflow in the upper airway [35,36], the governing equations for ALE description are as follows:

- Navier-Stokes equations
  (1) $$\frac{\partial u_{i}}{\partial t}-(u_{j}-v_{j})u_{i,j}-\gamma {\left(u_{i,j}+u_{j,i}\right)}_{,j}+\frac{1}{\rho^{2}}P_{,i}=g_{i}$$
  (2) $$u_{i,j}=0$$
- Mass conservation equation
  (3) $$\frac{\partial \rho }{\partial t}=w_{i}\frac{\partial \rho }{\partial x_{i}}-\rho \frac{\partial u_{i}}{\partial x_{j}}$$

In Equations (1)–(3), *u* denotes the particle velocity, v is the velocity of the mesh node, γ is the viscosity coefficient, ρ is the density of the fluid, *P* is the pressure, g is the acceleration due to gravity, and the relative velocity is w=u−v. The walls of the airway are assumed to be no-slip surfaces [37], $u_{i}=0$. The initial conditions are given by $u_{i}=u_{i}^{0}$ and $v_{i}=v_{i}^{0}$ with the initial velocity satisfying the incompressible condition of $u_{i}^{0}=0$.

The conservation equation of the solid field can be derived from Newton’s Second Law.

(4) $$\rho_{s}{\ddot{d}}_{s}=\nabla \cdot \sigma_{s}+f_{s}$$

where $\rho_{s}$ denotes the density of the solid, $\sigma_{s}$ is the Cauchy stress tensor, $f_{s}$ is the body force vector, and ${\ddot{d}}_{s}$ is the acceleration vector of the local solid field. At the fluid-solid coupling boundary, the stress and displacement obey

(5) $$\tau_{f}\cdot n_{f}=\tau_{s}\cdot n_{s}$$

(6) $$d_{f}=d_{s}$$

where $\tau_{f}$, $\tau_{s}$ and $n_{f}$, $n_{s}$ denote the stress and directional vectors of the fluid and solid, respectively. $d_{f}$ and $d_{s}$ denote the displacement of the fluid and of the solid, respectively.

## 3. Results

Simulations of a complete respiratory cycle in the upper airway, which consider two modes of breathing, i.e., eupnea and apnea, were conducted. The simulation data corresponded to the time at which the boundary pressure at the laryngeal outlet reached its peak value. The soft palate achieved maximum displacement and was extracted and used to investigate the displacement field of the soft tissues and the features of the fluid field within the airway for both eupnea and apnea.

### 3.1. One-Way Valve Effect of Soft Palate

**Figure 7:**
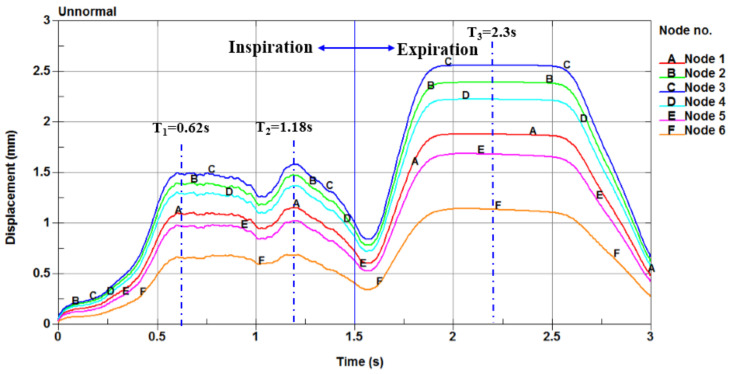
The displacement versus time curves of the six nodes in the soft palate of the OSAHS patient under apnea.

**Figure 8:**
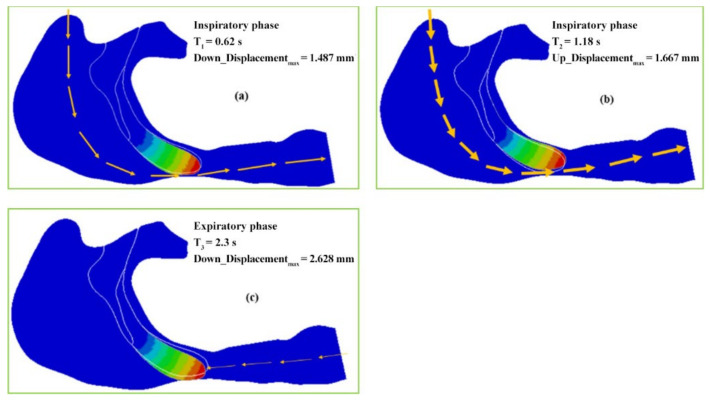
The maximum deformation and the one-way valve effect under apnea: (**a**) the soft palate moves to the posterior wall of the pharynx during inspiration phase, (**b**) the airway reopens during the inspiration phase, and (**c**) the airway is stenosed during expiration phase.

The displacement versus time curves at the six nodes in the soft palate of the OSAHS patient under apnea are illustrated in Figure 7. Figure 8 show the deformation conditions of the soft palate, where the maximum displacement occurs under apnea.

The locations of the six nodes are indicated in Figure 3. Figure 7 shows that the maximum displacements of the nodes in the expiration phase are much greater than those in the inspiration phase. In the inspiration phase, the maximum displacement occurs at T_2_ = 1.18 s and has a magnitude of 1.667 mm, at which point the soft palate moves to the anterior wall of the pharynx, as shown in Figure 8b. During the expiration phase (T = 1.8–2.6 s), the displacement of the soft palate fluctuates around the peak value (maximum displacement) with a magnitude of 2.628 mm; it occurs at T_3_ = 2.3 s and then the soft palate moves to the posterior wall of the pharynx, as shown in Figure 8c.

In Figure 8, the airflow is indicated by arrows. The arrows with thicker lines denote a better ventilation and a patent airway, and the arrows with thinner lines denote a lower ventilation and a narrower airway. The original position of the soft palate profile before a new cycle of respiration begins is plotted with a white line for the patient who is sleeping in a supine position under eupnea. Figure 8a shows that when T_1_ = 0.62 s, the soft palate moves to the posterior wall of the pharynx with a displacement of 1.487 mm, narrowing the airway and causing difficult passage of airflow during the inspiration phase. The impact of airflow is prone to cause vibrations of the soft palate and then the patient snores. Figure 8b shows that when T_2_ = 1.18 s, the soft palate moves to the anterior wall of the pharynx with a displacement of 1.667 mm. The airway reopens and the airflow can easily pass through the airway during the inspiration phase. Figure 8c shows that T_3_ = 2.3 s, the soft palate moves to the posterior wall of the pharynx with a maximum displacement of 2.628 mm during the expiration phase. The airway is stenosed and the airflow cannot pass with ease.

**Figure 9:**
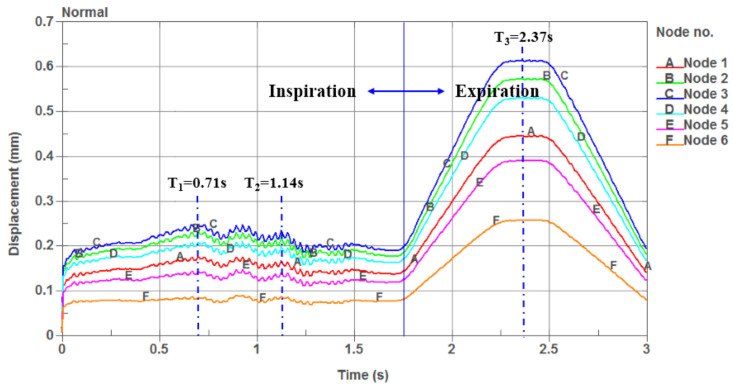
The displacement versus time curves of the six nodes in the soft palate of the OSAHS patient under eupnea.

**Figure 10:**
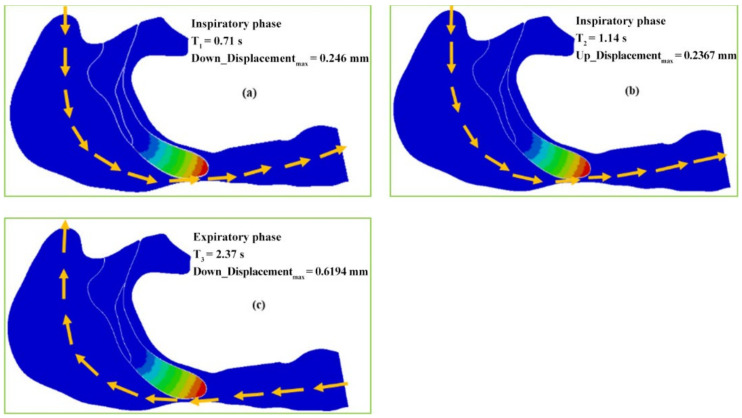
The maximum deformation of the OSAHS patient under eupnea: (**a**) the soft palate moves to the posterior wall of the pharynx during the inspiration phase, (**b**) the airway is more open during the inspiration phase, and (**c**) the airflow pass through the pharyngeal cavity during the expiration phase.

The displacement versus time curves at the six nodes in the soft palate of the OSAHS patient under eupnea are illustrated in Figure 9. Figure 10 show the deformation conditions of the soft palate, where the maximum displacement occurs under eupnea.

Figure 9 shows that the maximum displacement of the nodes in the phase of expiration is much greater than that in the inspiration phase. During the inspiration phase, the maximum displacement is 0.2778 mm and occurs at T_2_ = 1.14 s, at which point the soft palate moves to the anterior wall of the pharynx, as shown in Figure 10b. During the expiration phase, the displacement fluctuates around the peak value during the time interval T = 2.2–2.4 s, and the maximum displacement (0.6194 mm) occurs at T_3_ = 2.237 s, at which point the soft palate moves to the posterior wall of the pharynx, as shown in Figure 10c.

Figure 10a shows that the soft palate moves to the posterior wall of the pharynx with a displacement of 0.246 mm, and the airflow can easily pass through the airway during the inspiration phase at T_1_ = 0.71 s. Figure 10b shows that the soft palate moves to the interior wall of the pharynx with a displacement of 0.2367 mm at T_2_ = 1.14 s. The airway is opened more and the airflow can more easily pass through the airway during the inspiration phase. Figure 10c shows that when T_3_ = 2.37 s, the soft palate moves to the posterior wall of the pharynx with the maximum displacement of 0.6194 mm, and the airflow can still pass through the pharyngeal cavity at that time and go out from the nasal cavity during the expiration phase.

The magnitude of soft palate deformation tends to vary significantly for the OSAHS patient under different respiratory pressure conditions, which can be found in Figure 8 and Figure 10. Under the pressure condition of apnea, the deformation of the patient’s soft palate culminates in collapse, causing the airway obstruction. However, under the boundary conditions of eupnea, the airway of the patient merely undergoes stenosis, and collapse does not develop to the extent of a blockage.

Because the patient is lying in a supine position and is in a state of natural sleep, the soft palate already experienced a certain degree of retraction due to the effects of gravity. Comparison of the displacement–time curves shown in Figure 7 and Figure 9 with the deformation conditions shown in Figure 8 and Figure 10 shows that the displacement of the soft palate in the expiration phase is clearly greater than the displacement that occurs during inspiration. Thus, in the case of apnea, the deformation and collapse that lead to blockage of the airway occur during the expiration phase. Affected by the airflow during inspiration (T = 0–1.5 s), the soft palate initially displaces towards the posterior wall of the pharynx, causing the posterior region of the soft palate to become more constricted. The displacement reaches a peak at T_1_ = 0.62 s, and the soft palate then returns to the position it held prior to inspiration. A gradual movement of the soft palate towards the anterior wall of the pharynx then follows. The displacement reaches a peak of 1.667 mm at T_2_ = 1.18 s, and the change from inspiration to expiration occurs at T = 1.5 s. At that moment, the soft palate repeats its movement towards the posterior wall of the pharynx under the influence of counter directional flow. This is followed by a gentle increase in displacement until the displacement reaches a maximum value at T_3_ = 2.3 s.

In the expiration phase (T = 1.8–2.6 s), the soft palate maintains close contact with the posterior wall of the pharynx. It collapses during the expiration phase, blocks the airway, and obstructs the flow of air out of the pharynx. The displacement of the soft palate in the expiration phase is clearly greater than that during inspiration. In the case of apnea, the deformation and collapse lead to blockage of the airway during the expiration phase. In the case of eupnea, the movement of the soft palate follows a trajectory similar to that during apnea; however, the magnitude of the displacement in eupnea is 4–6 times smaller than that in apnea. Due to the rigidity of the airway, the soft tissue can withstand the pressure of the collapse in the eupnea state; therefore, the airway remains unobstructed throughout the respiration process.

It is evident that the pressure boundary conditions of respiration exert a sizable impact on the blockage of the airway. It is presumed that the change in the mechanical environment within the airway and the deformation of the soft tissues interact and influence one another. Particular attention should be on the one-way valve structure, which makes the soft palate more prone to collapse and causes blockage of the airway during the expiration phase under unusual airway pressure conditions.

### 3.2. Mechanical Characteristics of the Fluid Field in the Upper Airway

#### 3.2.1. The Pressure Field of the Cavity

As the OSAHS patient has a hypertrophic soft palate, the pharynx proximal to the soft palate becomes constricted. This can lead to an accelerated change in upper airway pressure during sleep and affect the clear passage of the upper airway. The high pressure difference of the fluid field acts on the soft palate and produces considerable displacement. Concurrent to these events, the inner pressure of the upper airway would continue to decrease. This would make the soft palate collapse and result in partial or even full closure of the upper airway, causing snoring and apnea.

**Figure 11:**
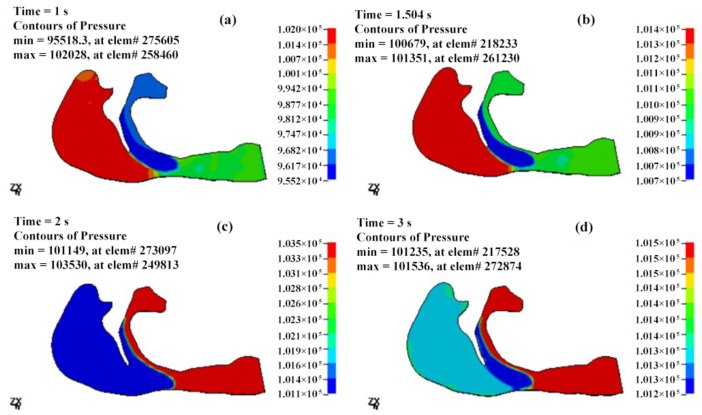
The pressure distributions of the fluid field of the OSAHS patient’s upper airway under the state of apnea (pressure unit in Pa). (**a**,**b**) The inspiration phase; (**c**,**d**) the expiration phase.

**Figure 12:**
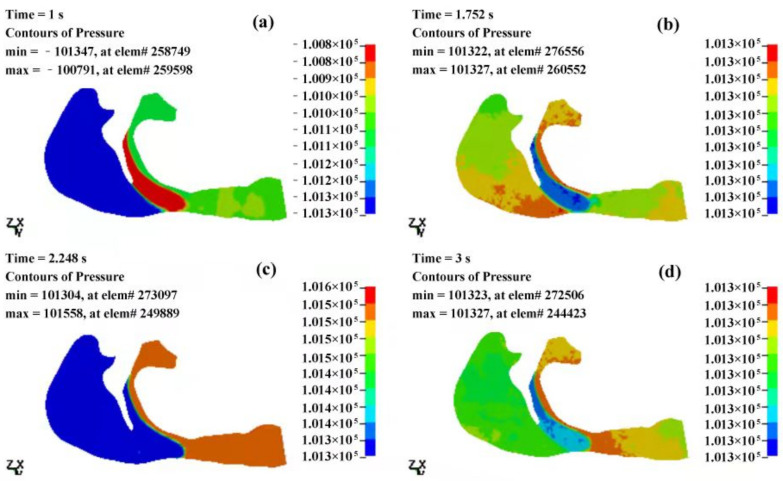
The pressure distributions of the fluid field of the OSAHS patient’s upper airway under the state of eupnea (pressure unit in Pa). (**a**,**b**) The inspiration phase; (**c**,**d**) the expiration phase.

The pressure distributions of the OSAHS patient’s fluid field during apnea and eupnea are depicted in Figure 11 and Figure 12. The data show the cavity pressure change in the OSAHS patient’s upper airway in the states of eupnea and apnea are tabulated in Table 1 and Table 2, respectively. By comparing Figure 11 and Figure 12 with Table 1 and Table 2, the following findings can be observed.

**Figure 13:**
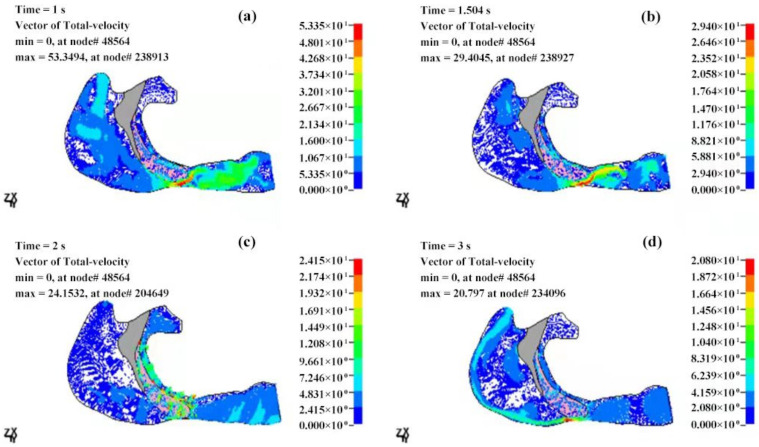
The velocity distributions of the fluid field of the OSAHS patient’s upper airway under the state of apnea (velocity unit in m/s). (**a**,**b**) The inspiration phase; (**c**,**d**) the expiration phase.

**Figure 14:**
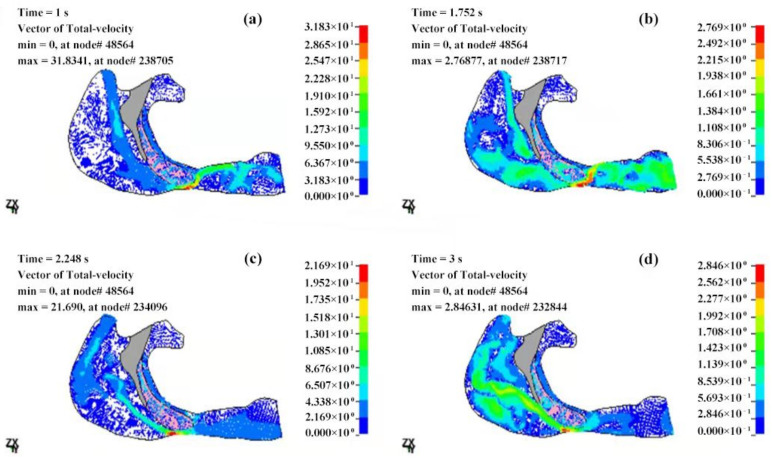
The velocity distributions of the fluid field of the OSAHS patient’s upper airway under the state of eupnea (velocity unit in m/s). (**a**,**b**) The inspiration phase; (**c**,**d**) the expiration phase.

At 1 s after the beginning of inspiration, the decrease in the inspired flow volume, velocity and pressure reach the maximum value. In the case of apnea, the measured pressure at the anterior of the nostrils is 102,028 Pa, as shown in Figure 11a and Table 1. There is a pressure difference of −2495 Pa (Table 1) within the entire cavity, and the maximum measured speed of the airflow is 53.35 m/s (Figure 13a). However, in the case of eupnea, the pressure difference at the lower cavity is −302 Pa (Table 2), and the maximum speed of the inspired airflow is 31.83 m/s (Figure 14a).

In the case of apnea, at the time of T = 1.5 s, the respiration changes from inspiration to expiration, and the low pressure in the vicinity of the soft palate begins to increase. The pressure at the anterior nostrils is 101,351 Pa (Figure 11b), the pressure difference of the entire cavity is −321 Pa (Table 1), and the maximum measured speed of the expired airflow is 29.4 m/s (Figure 13b). During the expiration phase (T = 1.5–3 s), the location of the highest pressure in the upper airway cavity begins to move towards the laryngopharynx and oropharynx, while the lowest pressure occurs at the posterior wall of the pharynx close to the soft palate.

In the case of eupnea at time point T = 1.75 s, the inspiration switches to expiration. There is a −98 Pa (Table 2) difference inside the cavity, and the maximum measured speed of the expired airflow is 27.69 m/s (Figure 14b). In the case of apnea, during the expiration phase at T = 2 s, the pressure difference in the cavity is 2099 Pa (Table 1), and the maximum speed of the expired airflow is 24.15 m/s (Figure 13c). In the case of eupnea at T = 2.25 s, the pressure difference inside the cavity is 99 Pa (Table 2), and the maximum speed of the expired airflow is 21.69 m/s (Figure 14c).

Regardless of if the patient is in a state of eupnea or apnea, there is a significant pressure difference between the anterior and the posterior part of the soft palate during expiration. The apnea experiences a higher pressure than the eupnea, and this causes the soft palate to move towards the posterior pharyngeal wall. According to the mechanical characteristics of the fluid field of the airway in the states of eupnea and apnea, it is presumed that the conditions of the airway blockage throughout the complete respiratory cycle would determine the gradient of pressure decrease within the airway. The more severe the blockage, the higher the pressure gradient.

#### 3.2.2. Velocity Field of the Cavity

The velocity distributions of the fluid field of the OSAHS patient’s upper airway in the states of apnea and eupnea are shown in Figure 13 and Figure 14. It is observed that the distribution of the streamlines is similar in the states of eupnea and apnea during the inspiration phase. From the velocity streamline graph of the cavity interior, it can be observed that, when the airflow enters the pharyngeal cavity from the nasopharynx, it is directed from the horizontal to the vertical direction and impacts the posterior wall of the pharynx.

At the nasopharynx, the airflow enters the pharyngeal cavity from the nasal cavity. The resulting airflow distribution is uneven with slightly higher airflow speed near the hard palate than that at the top of the nasopharynx, where turbulent flow occurs from time to time, and the airflow is deflected downwards. In the pharyngeal cavity, the direction of the airflow is mostly perpendicular to the sagittal plane, moving from the rear wall to the front wall, and this has an impact on the soft palate. Within the cavity, the fluid primarily undergoes laminar flow with minor turbulent flow in local areas.

However, there is a significant difference in eupnea and apnea. The airflow is directed from the laryngeal cavity to the pharyngeal cavity during the expiration phase. There are significant signs of turbulent flows in the laryngeal cavity. These flows gradually move in a vertical direction towards the posterior pharyngeal wall. Given that the soft palate maintains secure contact with the posterior pharyngeal wall under the effects of the airflow, collapse followed by blockage could occur in the airway. When the pressure starts decreasing slowly and the posterior part of the soft palate begins to recover from a state of stenosis, turbulent flows would be evident in the laryngeal cavity again and the direction of the airflow would change from vertical to horizontal. Therefore, air would pass the posterior pharyngeal wall and exit from the front of the nostrils.

In the state of eupnea and after the transition from inspiration to expiration, there is a persistent state of turbulence. After that the airflow in the direction of the pharyngeal cavity is at an angle of 45–60 degrees to the posterior pharynx wall, which impacts the wall along with the soft palate before exiting from the anterior part of the nostrils through the pharyngeal cavity.

At different moments of eupnea and apnea, during respiration, the velocity distribution of the airflow in the upper airway is low at both ends and high in the middle. In this study, the CT data of an OSAHS patient in natural sleep are used to construct the FE model. Furthermore, the condition of apnea is taken into account. When clinically acquired time-dependent pressure boundary conditions in the cavity are applied, the airway collapses under the extreme negative pressure. This results in airway stenosis that significantly increases the airflow speed.

It should be noted that the calculated airflow velocity values in this paper are different to the results in the literature, in which the maximum airflow velocity value in upper airway of the OSAHS patient calculated by Jeong et al. [38] was 12.19 m/s. The maximum airflow velocity value in upper airway, of the OSAHS patient of Sung et al. [39], obtained was 15.14 m/s. In the study carried out by Tan et al. [8], the maximum calculated airflow velocity values were 19.26 ± 12.4 m/s and 19.46 ± 13.1 m/s. The reason is that most of the previous studies used quantitative flow boundary, resulting in minor change of the airflow in the upper airway but the time-varying pressure boundary conditions obtained clinically have been used in this study. Different from the case of calm breathing considered in the literature, when considering the extreme pressure boundary condition in laryngeal cavity during apnea, the airflow in the upper airway changes dramatically with extreme time-varying negative pressure. In extreme mechanical conditions, such as coughing or sneezing, the airflow rate in the airway is very different from that of normal breathing. According to Bernoulli, a narrower airway would have faster the airflow. Beni et al. [40] pointed out that when person sneezes, the velocity at nasopharynx increases by up to 125 m/s, so that the cross-section changing in this area leads the fluid acts as a jet flow.

## 4. Discussions

As a patient’s body experiences a change in physiological conditions or in the external environment, the mechanical properties of the upper airway, the surrounding tissues, and the mechanical boundary conditions of respiration change correspondingly. This would result in mechanical behavior disorders and obstruction of the upper airway. The biomechanical mechanism plays an important role in the occurrence of upper airway obstruction. By integrating medical image processing and monitoring the data in the 3D FE model, computational biomechanics, the theory of modern otorhinolaryngology, biomechanical simulation was regarded as the state-of-the-art technique in the study of the pathogenesis of OSAHS.

In the current study, CT-scanned data obtained from an OSAHS patient during natural sleep was used to build a numerical model for biomechanical simulation. Using the clinical monitoring data to model the boundary conditions and considering the influence of gravity on the soft palate while the patient sleeps in a supine position, the numerical simulation results were able to provide a satisfactory representation of the patient’s actual respiratory physiological process during sleep. Two breathing patterns, eupnea and apnea, of the OSAHS patient, sleeping naturally in a supine position, were simulated. The main conclusions of this research are summarized below.

- The airflow characteristics during the inhalation phase are noticeably different from those in the exhalation phase for both eupnea and apnea. As the OSAHS patient breathes, the minimum pressure occurs alternately between the soft palate and the anteroposterior wall of pharynx, which causes the soft palate to vibrate during respiration, and therefore, the patient snores in sleep.
- The soft palate in pharyngeal cavity would exerts a one-way valve effect and collapses in the exhalation phase if the patient sleeps in the supine position, regardless of eupnea or apnea.
- The mechanical environment of the airway is directly dependent on the action of the airflow. If the mechanical properties of the soft palate remain unchanged, the pressure makes the soft palate collapse in apnea. In eupnea, the pressure allows the airflow to pass freely through the airway.

However, the upper airway is a 3D biomechanical structure characterized as being complex, delicate, and physiologically self-adaptive. OSAHS can occur due to the factors of obstruction or due to poor ventilation. Therefore, it is necessary to establish a biomechanical model of the upper airway, especially the upper airway of OSAHS patients, to further study the mechanisms of obstruction and collapse of the upper airway. Based on the clinically obtained data and the numerical simulations, the results of the OSAHS patient’s airflow velocity during natural sleep provide a clinical reference value.

With a quasi-3D FE model of the OSAHS patient’s upper airway, the sagittal deformation of the upper airway was investigated in the current study. The factors that affect the collapse of the airway have been analyzed. The results showed that the one-way valve effect of the soft palate in the pharyngeal cavity is one of the important mechanical factors that causes collapse of the upper airway. The results of biomechanical simulation mutually confirm the one-way valve anatomical structure of the soft palate and its physiological and pathological effects. Based on the calculated results combined with the clinical data, we conducted a more in-depth study on the pathogenesis of OSAHS from a multidisciplinary perspective, which is helpful to improve existing methods and develop better methods for the diagnosis and treatment of OSAHS.

In future studies, monitoring the actual airflow velocity in the OSAHS patients’ upper airway during natural sleep period by installing velocity sensors and using other methods in clinical trials is expected. In this study, the linear elastic modulus was adopted for the soft palate in calculations. In fact, the constitutive relation of soft tissues, such as soft palate, is nonlinear. Therefore, we will consider the nonlinear constitutive relation of soft tissue in subsequent research. It is also important to establish an advanced and sophisticated 3D numerical model of OSAHS patient upper airways, to provide more realistic simulations for studying the mechanisms of upper airway collapse in the future.

## Figures and Tables

**Table 1:** The change of the cavity pressure of the OSAHS patient under apnea.

T (s)	*P*_max_ (Pa)	*P*_min_ (Pa)	Δ*P* (Pa)	Pressure Drop (Pa)
0.5	101,408	98,943	2465	−1301
1	102,028	95,518	6510	−2495
1.25	101,306	98,671	2635	−1712
1.5	101,351	100,679	672	−321
1.75	102,387	101,189	1198	1010
2	103,530	101,149	2381	2099
2.5	103,034	101,184	1850	1591

Note: *P*_max_ and *P*_min_ are the maximum and minimum pressures in cavity. Pressure difference in cavity $\Delta P=P_{\max}-P_{\min}$. Pressure drop is the pressure difference between the inlet and outlet of the upper airway.

**Table 2:** The change of the cavity pressure of the OSAHS patient under eupnea.

T (s)	*P*_max_ (Pa)	*P*_min_ (Pa)	Δ*P* (Pa)	Pressure Drop (Pa)
0.5	101,332	100,945	387	−201
1	101,337	100,709	628	−302
1.25	101,333	100,960	373	−201
1.5	101,329	101,127	202	−98
1.75	101,327	101,321	6	1
2	101,429	101,258	171	99
2.5	101,533	101,225	308	198

## Data Availability

Not applicable.
